# Clinicopathological features and outcomes of immature PIT-1 lineage tumors: A high-risk pituitary neuroendocrine tumor/pituitary adenoma subtype per WHO 2022

**DOI:** 10.1007/s11102-026-01691-9

**Published:** 2026-05-13

**Authors:** Aysel Unver Ozkahraman, Aslihan Pekmezci, Huseyin Karatay, Ebubekir Akpinar, Buruc Erkan, Mehmet Karagulle, Burak Kocak, Mutlu Niyazoglu, Esra Hatipoglu

**Affiliations:** 1 Division of Endocrinology, Department of Internal Medicine, University of Health Sciences, Basaksehir Cam and Sakura City Hospital, Istanbul, Turkey; 2 Department of Pathology, University of Health Sciences, Basaksehir Cam and Sakura City Hospital, Istanbul, Turkey; 3 Department of Neurosurgery, University of Health Sciences, Basaksehir Cam and Sakura City Hospital, Istanbul, Turkey; 4 Department of Radiology, Basaksehir Cam and Sakura City Hospital, Istanbul, Turkey; 5 Pituitary Disorders Application and Research Center (HATMER), University of Health Sciences, Aysel Unver Ozkahraman, Istanbul, Turkey

**Keywords:** Immature PIT-1 lineage tumor, Pituitary neuroendocrine tumor, WHO 2022 classification, Transcription factor, Aggressive pituitary tumor, Intracranial seeding

## Abstract

**Purpose:**

Immature PIT-1 lineage tumors are classified as high-risk pituitary adenomas/pituitary neuroendocrine tumors (PitNETs) under the 2022 WHO Classification, yet clinical outcome data remain scarce. This study aimed to characterize clinicopathological features and treatment outcomes of this rare entity.

**Methods:**

We retrospectively analyzed 13 patients with pathologically confirmed immature PIT-1 lineage tumors who underwent surgery at our tertiary center between January 2022 and December 2024. Diagnoses were established according to WHO 2022 criteria using comprehensive transcription factor (TF) immunohistochemistry. Clinical, radiological, histopathological, treatment, and follow-up data were evaluated.

**Results:**

Immature PIT-1 lineage tumors accounted for 4% (21/525) of surgically treated pituitary adenomas, of whom 13 with complete follow-up data constituted the primary analysis cohort. Median age was 37 years (range: 25–73), with female predominance (61.5%). Notably, 61.5% presented with hormonal hypersecretion: acromegaly (38.5%), TSH-secreting tumors (15.4%), and GH-PRL co-secretion (7.7%). All patients had macroadenomas (median diameter 28 mm (range: 15–59 mm)); 38.5% demonstrated cavernous sinus invasion (Knosp grades 3–4). Immunohistochemically defined plurihormonal phenotype was present in 77% of cases; cytologic atypia was identified in all cases. Despite maximal surgical resection, residual disease persisted in 46% and 31% required reoperation at 16-month follow-up (range: 4–51 months). The median Ki-67 proliferation index was 4% (IQR: 2–10%; range: 1–35%), and the median mitotic count was 4 per 2 mm² (IQR: 1–8; range: 1–20).

**Conclusion:**

Immature PIT-1 lineage tumors exhibit aggressive behavior with high residual disease rates, with 31% of patients requiring reoperation, 23% receiving adjuvant radiotherapy, and somatostatin analogue resistance observed in two patients. Routine TF immunohistochemistry is essential for accurate diagnosis; management requires maximal safe surgical resection and intensive long-term surveillance.

**Supplementary Information:**

The online version contains supplementary material available at 10.1007/s11102-026-01691-9.

## Introduction

 Pituitary adenomas are the third most commonly occurring primary brain tumors and form a substantial majority of all sellar masses [1]. Pituitary adenomas, previously termed pituitary adenomas in earlier classifications, generally originate from the anterior pituitary and are typically low-grade tumors, although in rare cases, they can exhibit aggressive behavior.

Pituitary adenomas are classified according to their transcription factor (TF) and cell differentiation. TFs are indicative of particular cell lineages that subsequently generate the terminally differentiated cellular phenotypes observed in pituitary adenomas. Corticotropic tumors arise from T-box family member TBX19 (T-PIT) lineage, gonadotropic tumors originate from steroidogenic factor-1 (SF-1) lineage. The pituitary transcription factor 1 (PIT-1) lineage encompasses diverse cellular subtypes (lactotropes, somatotropes, thyrotropes, and their plurihormonal variants), with immature PIT-1 tumors representing the least differentiated form. The 2022 WHO update mandates the use of histological subtypes for determining aggressiveness, replacing traditional prognostic scales like stage or invasion alone. This paradigm shift emphasizes TF lineage and cellular differentiation and categorizes certain subtypes, including immature PIT-1 lineage tumors, as having “high risk of adverse clinical outcomes” [2]. For these tumors, risk assessment combines histological classification with radiological invasion (Knosp/Hardy grading) and proliferative markers (Ki-67, p53) [2, 3].

Immature PIT-1 lineage tumors are histologically characterized by chromophobic to amphophilic, poorly differentiated cells that lack terminal differentiation, often displaying variable pleomorphism, nuclear atypia, macronucleoli, and nuclear pseudoinclusions. Immunohistochemically, they are consistently positive for PIT-1 and show variable staining with one or more PIT-1-related hormones (growth hormone (GH), prolactin (PRL) and thyroid-stimulating hormone (TSH)). They may clinically present as silent, but they can also manifest with acromegaly, hyperprolactinemia, or central hyperthyroidism [2]. Although uncommon, it is critical to recognize this subset of tumors owing to its marked distinctions from more mature PIT-1 lineage forms and its predisposition to an aggressive phenotype. Recent dedicated series, although limited to small cohorts of 6 to 15 cases, consistently demonstrate significant aggressiveness, with cavernous sinus invasion rates of 40–50% and a 10-year recurrence risk of up to 50%, markedly higher than those observed in mature PIT-1 lineage counterparts [4–6]. Consequently, the scientific literature contains limited information about this subgroup, especially concerning their clinical outcomes, with the largest dedicated series including limited number of cases.

This study sought to characterize the clinicopathological features and treatment outcomes in a consecutive cohort of 21 patients with pathologically confirmed immature PIT-1 lineage tumors diagnosed under the WHO 2022 criteria, of whom 13 received longitudinal follow-up care at our institution and constitute the primary analysis cohort. In addition to histopathological characterization, this study provides detailed longitudinal clinical outcome data, including treatment responses, reoperation rates, medical therapy resistance, and postoperative hormonal outcomes, which have been underrepresented in prior series.

## Materials and methods

This study was conducted at Basaksehir Cam and Sakura City Hospital between January 2022 and December 2024. Patients admitted to our hospital during this period were identified, and their retrospective data were collected and integrated with follow-up information. A total of 525 patients underwent pituitary surgery during this period. Pathological examination showed results consistent with immature PIT-1 lineage pituitary adenomas in 21 (4%) of these patients.

Eight cases (38%) were excluded from the primary analysis cohort due to loss to follow-up or transfer to external institutions for long-term care. Further analysis included 13 patients who received follow-up care at our hospital’s endocrinology clinic. Clinical data, laboratory and endocrinologic findings, pathologic characteristics, radiologic outcomes, and follow-up details were retrospectively retrieved from the patients’ medical records.

To assess potential selection bias, baseline clinicopathological characteristics (age, sex, tumor size, Knosp grade, hormonal phenotype, Ki-67 index) were compared between the analyzed cohort (*n* = 13) and excluded patients (*n* = 8) using chi-square test for categorical variables and Mann-Whitney U test for continuous variables (Supplementary Table 1). No statistically significant differences were observed (all *p* > 0.05), suggesting the follow-up cohort is representative of the overall immature PIT-1 population at our center.

The study was conducted in accordance with the ethical principles established in the Declaration of Helsinki and was approved by the hospital’s ethics committee (approval number: 194, dated August 28, 2024).

### Histopathological examination

The diagnosis of immature PIT-1 lineage pituitary adenomas was established in the pathology laboratory of our tertiary care center by endocrine pathologists with expertise in pituitary pathology according to the 2022 WHO classification of pituitary tumors. Hematoxylin–eosin-stained (H&E) sections were examined for architectural pattern, reticulin framework, cellular pleomorphism, cytologic atypia (including macronucleoli and nuclear pseudoinclusions), mitotic activity, and other features of aggressiveness.

Immunohistochemical studies included pituitary TFs PIT-1, SF-1, and TPIT, as well as estrogen receptor alpha (ERα) and GATA-3, together with pituitary hormones GH, PRL, TSH, follicle-stimulating hormone (FSH), luteinizing hormone (LH), and adrenocorticotropic hormone (ACTH), in line with current WHO recommendations. The Ki-67 (MIB-1) labeling index was determined in proliferative hot spots. Mitotic figures were counted in a standardized area of 2 square millimeters (mm²). The presence of fibrous bodies was assessed on H&E and, when necessary, confirmed using low-molecular-weight cytokeratin (CK18) immunostaining.

Immature PIT-1 lineage pituitary adenomas/PitNETs were defined as PIT-1–positive, SF-1– and TPIT-negative, showing limited and often heterogeneous expression of PIT-1–dependent hormones (GH, PRL, and/or TSH), with or without GATA-3 expression, and displaying characteristic immature chromophobic or amphophilic cytology with marked cytologic atypia, macronucleoli, and nuclear pseudoinclusions, consistent with the 2022 WHO criteria for this entity.

### Endocrinological evaluation

Patients’ preoperative, follow-up and the latest hormone profiles (GH, Insulin-like Growth Factor-1 (IGF-1), PRL, TSH, free T4 and free T3 (fT4, fT3), ACTH, cortisol) were recruited from their clinical registries.

For acromegaly, biochemical remission was defined according to the 2024 Pituitary Society consensus criteria: random serum GH < 1.0 µg/L, nadir GH during oral glucose tolerance test (OGTT) < 0.4 µg/L, and normalization of serum IGF-1 to age- and sex-matched reference ranges [7]. For TSH-secreting pituitary adenomas, remission was defined as normalization of serum free T4 and free T3 levels with appropriately suppressed or normal TSH concentrations, in accordance with established diagnostic criteria. Patients with persistently elevated hormone levels or detectable residual tumor on postoperative MRI were considered to have residual disease [8]. For clinically non-functioning pituitary adenomas, remission was defined as the absence of radiologically detectable residual or recurrent tumor on postoperative MRI, consistent with the Pituitary Society international consensus recommendations [9].

Recurrence was defined as either: (1) Radiological recurrence: New measurable tumor in patients with gross total resection (2) Biochemical recurrence: Re-elevation of previously normalized hormone levels.

Follow-up protocol: Patients underwent clinical evaluation, hormonal assessment, and pituitary MRI at 3–6 months postoperatively, then annually for the first 3 years, and every 1–2 years thereafter, or more frequently if clinically indicated.

### Pituitary MRI

Preoperative, postoperative, and current pituitary MRI examinations were evaluated by an experienced neuroradiologist. All pituitary MR imaging examinations were conducted with a 3 T MR imaging unit (Ingenia; Philips Healthcare). The imaging protocol for each patient comprised coronal and sagittal spin-echo T1WI, coronal and sagittal spin-echo T2WI, coronal dynamic, contrast-enhanced T1WI, and conventional coronal and sagittal late contrast-enhanced T1WI sequences following the administration of gadolinium-based contrast agents. For each examination, 0.1mmol/kg of gadolinium-based contrast agent was administered. Pituitary MR imaging protocols complied with recommendations of the European Society of Endocrinology guidelines for the management of aggressive pituitary tumors and carcinomas [10]. The following radiological features were documented on preoperative MRI: the tumor’s largest dimension (selected from the three dimensions in the patients’ medical records), relationship with the optic chiasm, suprasellar extension, infrasellar extension with or without sphenoid sinus involvement, cavernous sinus invasion graded according to the Knosp classification system, and MRI T2A image signal intensities. Postoperative and follow-up MRI scans were reviewed for residual tumor and recurrence assessment.

### Visual field examination

Visual field examination was performed on patients with macroadenomas or tumors in close proximity to the optic chiasm. Postoperative visual field examination was performed on those who showed defects in the preoperative test. Visual field testing was conducted using the Humphrey Visual Field Analyzer 24 − 2 SITA Standard protocol (Carl Zeiss Meditec, Dublin, CA, USA, 2019).

### Statistical analysis

Statistical analyses were conducted using SPSS software, version 25.0. Independent categorical variables underwent evaluation via the chi-square test. Qualitative variables were presented as absolute numbers and percentages. For continuous variables, sample distribution was assessed using the Kolmogorov-Smirnov test. For variables that followed a normal distribution, descriptive statistics comprised the mean and standard deviation, but for those not normally distributed, the median, interquartile range (IQR), and range (minimum–maximum) were utilized.

## Results

### Basal characteristics and preoperative evaluation

In this cohort, immature PIT-1 lineage tumors made up 4% (21 of 525) of the total pituitary adenomas spectrum. Table 1 provides a summary of the demographic, follow-up analysis, and clinical data for the 13 cases included in this study.

**Table 1:** Baseline demographic and clinical features of the study population

	Total (*n* = 13)
Current age, median age (range) (years old)	40 (28–73)
Gender, n (%)	
Male	5 (38.5)
Female	8 (61.5)
Age at diagnosis, median age (range) (years old)	37 (25–73)
Follow-up duration, median (range) (months)	16 (4–51)
Initial presenting symptom, n (%)	
Headache	4 (30.8)
Visual defect	3 (23.1)
Incidental	2 (15.4)
Menstrual irregularity	2 (15.4)
Hearing loss	1 (7.7)
Acromegalic appearance	1 (7.7)
Main biochemical findings before surgery, n (%)	
Acromegaly	5 (38.5)
Non-functioning	5 (38.5)
Central hyperthyroidism	2 (15.4)
GH-PRL co-secretion	1 (7.7)
Preoperative pituitary hormone deficiencies, n (%)	
Central adrenal insufficiency	3 (23.1)
Central hypothyroidism	2 (15.4)
Central hypogonadism	3 (23.1)
Central adrenal insufficiency + hypothyroidism	1 (7.7)
Preoperative visual field testing, n (%)	
Normal	8 (61.5)
Deficit	5 (38.5)
Monocular blindness	1
Monocular blindness + contralateral temporal hemianopia	1
Unilateral temporal hemianopia	3

Abbreviations: GH, growth hormone; TSH, thyroid-stimulating hormone; PRL, prolactin

Among patients’ first visits to an outpatient clinic, headache was the predominant complaint (30.8%), followed by visual disturbance (23.1%), menstrual irregularity (15.4%), and acromegalic features (7.7%). Two patients (15.4%) were diagnosed incidentally

GH hypersecretion was present in 6 cases (46.2%), comprising 5 cases (38.5%) with isolated GH elevation and 1 case (7.7%) with concomitant GH and PRL secretion. Two patients (15.4%) displayed elevated TSH levels with concomitantly elevated free T4 and free T3, consistent with central hyperthyroidism. An isolated and mild increase in PRL was noted in one instance, which was ascribed to pituitary stalk compression rather than actual PRL hypersecretion. Preoperative hypopituitarism was observed in 53.8% of cases (7/13), including hypogonadism in 3 patients (23.1%), adrenal insufficiency in 3 patients (23.1%), and central hypothyroidism in 2 patients (15.4%), with one patient (7.7%) demonstrating concurrent adrenal insufficiency and central hypothyroidism. Preoperatively, visual field tests demonstrated defects in 38.5% of the cases.

The median follow-up duration was 16 months (range: 4–51 months), with 5 patients (38.5%) achieving ≥ 24 months and 3 patients (23.1%) achieving ≥ 36 months of follow-up.

###  Radiological findings

Preoperative pituitary MRI studies consistently revealed macroadenomas in all 13 patients, with a median maximal diameter of 28 mm (IQR: 16–41 mm; range: 15–59 mm). T2-weighted imaging demonstrated hyperintense signals in 8 cases (61.5%), isointense signals in 3 cases (23.1%), hypointense signal in 1 case (7.7%), and mixed signal intensity in 1 case (7.7%) reflecting heterogeneous tumor composition.

Cavernous sinus invasion, assessed using the Knosp classification, was present in 5 cases (38.5%; Knosp grades 3–4), while 8 cases (61.5%; Knosp grades 1–2) showed no definitive invasion. Notably, no tumors were classified as Knosp grade 0 (no contact with cavernous sinus).

Tumor extension patterns revealed suprasellar extension in 12 cases (92.3%) and infrasellar extension in 10 cases (76.9%), with concurrent bidirectional extension in 9 cases (69.2%). The detailed radiological characteristics of each case are summarized in Table 2.

**Table 2:** Preoperative radiological assessment of the cohort

Patient number	Maximal diameter of tumor	KNOSP	MRI T2W signal intensity	Suprasellar extension	Infrasellar extension
1	27 mm	3	isointense	yes	yes
2	21 mm	1	hyperintense	yes	yes
3	17 mm	2	hypointense	no	yes
4	30 mm	1	hyperintense	yes	yes
5	15 mm	2	isointense	yes	yes
6	15 mm	1	isointense	yes	no
7	39 mm	3	hyperintense	yes	yes
8	43 mm	4	hyperintense	yes	yes
9	15 mm	2	hyperintense	yes	yes
10	57 mm	4	hyperintense	yes	no
11	59 mm	2	hyperintense	yes	no
12	40 mm	4	hyperintense and hypointense	yes	yes
13	28 mm	2	hyperintense	yes	yes

**Abbreviations: MRI**, magnetic resonance imaging; **T2W**, T2-weighted imaging; **mm**, millimeters

### Histopathological analysis

The principal histomorphological observations and the data yielded by immunohistochemical analysis are summarized within Table 3.

**Table 3:** Detailed immunohistochemical profile and proliferative activity of the study cohort

Patient number	PIT-1	GATA-3	ER	GH	PRL	TSH	Ki-67	Mitosis (per 2 mm²)	CK-18	Fibrous bodies
**1**	**+**	focal	patchy	sparse	**-**	**-**	35	20	focal	no
**2**	**+**	focal	**-**	**-**	sparse	**-**	1	1	focal	yes (20%)
**3**	**+**	**-**	**-**	diffuse	diffuse	**-**	5	6	focal	yes (30%)
**4**	**+**	**-**	**-**	patchy	patchy	sparse	2	1	focal	**-**
**5**	**+**	**-**	focal	patchy	diffuse	**-**	6	5	diffuse	yes (80%)
**6**	**+**	**-**	-	focal	focal	focal	4	4	focal	**-**
**7**	**+**	**-**	focal	sparse	sparse	**-**	1	1	focal	yes (30%)
**8**	**+**	focal	**-**	sparse	sparse	**-**	5	7	diffuse	**-**
**9**	**+**	**-**	**-**	focal	focal	**-**	2	1	**-**	**-**
**10**	**+**	patchy	**-**	**-**	focal	**-**	2	1	**-**	**-**
**11**	**+**	diffuse	**-**	**-**	diffuse	focal	15	8	**-**	**-**
**12**	**+**	**-**	**-**	diffuse	patchy	**-**	15	12	focal	yes (10%)
**13**	**+**	sparse	focal	sparse	sparse	sparse	2	1	focal	yes (20%)

**Abbreviations: PIT-1**, pituitary-specific positive transcription factor 1; **GATA-3**, GATA binding protein 3; **ER**, estrogen receptor; **GH**, growth hormone; **PRL**, prolactin; **TSH**, thyroid-stimulating hormone; **CK-18**, cytokeratin 18; mm², square millimeters; **(+)**, positive; **(−)**, negative

Semiquantitative immunohistochemical expression was graded as follows: Sparse, 1–10% of tumor cells; Focal, 11–30%; Patchy, 31–70%; Diffuse, > 70%

All tumors demonstrated uniform PIT-1 nuclear positivity (100%) and were consistently negative for T-PIT, SF-1, ACTH, LH, and FSH, confirming pure PIT-1 lineage differentiation.Variable immunoreactivity for at least one PIT-1-related hormone (GH, PRL, TSH) was universally present. Of the 13 cases, 10 (76.9%) exhibited a plurihormonal phenotype. GH and PRL co-expression was the most common pattern, observed in 6 cases (46.2%), while tri-hormonal positivity (GH, PRL, and TSH) was noted in 3 cases (23.1%).

GATA-3 positivity was observed in 6 cases (46.2%), and ER positivity in 4 cases (30.8%). Cytokeratin-18 (CK-18) expression was present in 10 tumors (77%), with fibrous bodies identified in 6 cases (46%), occupying a median of 20% of tumor volume (range: 10–80%). Cytologic atypia, a defining characteristic of immature PIT-1 tumors, was universally present, manifesting as macronucleoli, nuclear pseudoinclusions, and chromophobic or amphophilic cytoplasm.

The median Ki-67 proliferation index was 4% (IQR: 2–10%; range: 1–35%), with 7 patients (53.8%) demonstrating Ki-67 ≥ 3%, a threshold associated with increased proliferative activity. The median mitotic count was 4 per 2 mm² (IQR: 1–8; range: 1–20).

**Fig. 1:**
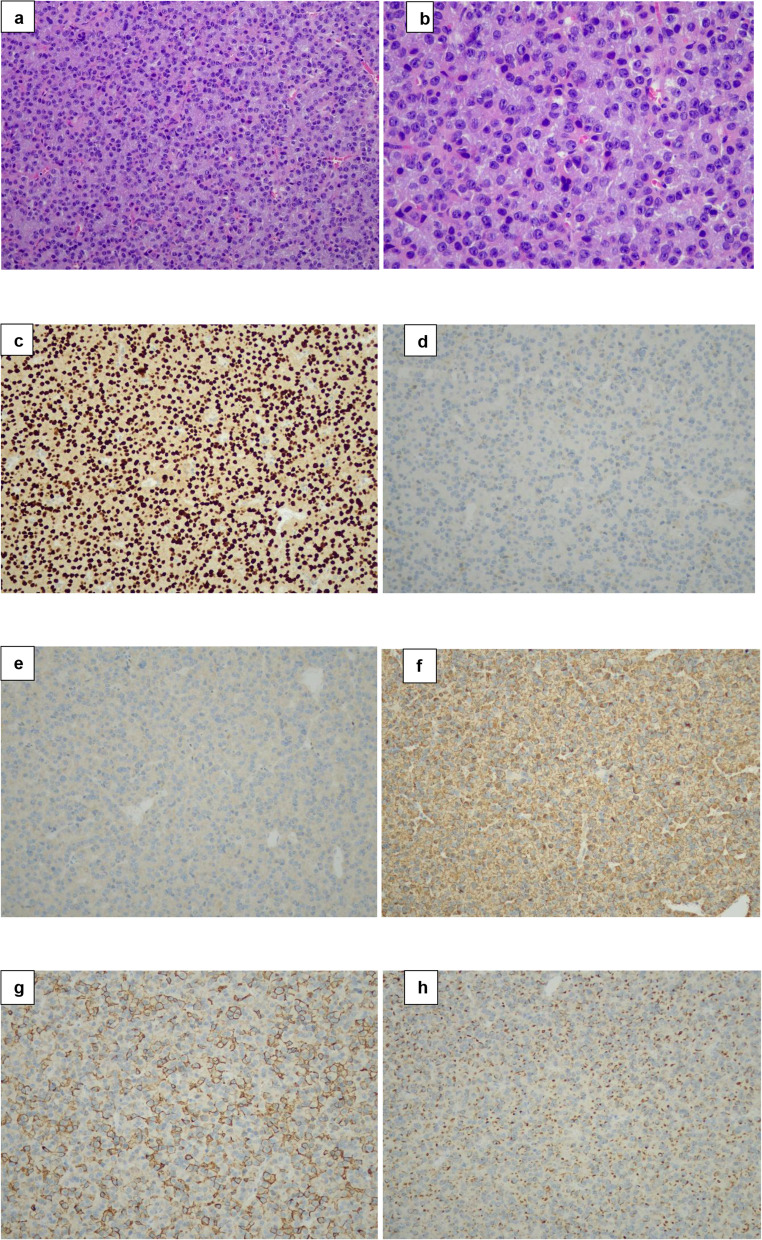
Representative histopathological and immunohistochemical features of Case 3. (**a**) Hematoxylin and eosin (H&E) (×20): solid growth pattern composed of tumor cells with pale eosinophilic to amphophilic cytoplasm. (**b**) H&E (×40): moderate nuclear pleomorphism with conspicuous nucleoli. (**c**) Pituitary-specific positive transcription factor 1 (PIT-1) immunostain (×20): diffuse nuclear positivity. (**d**) Estrogen receptor (ER) immunostain (×20): negative. **(e)** GATA binding protein 3 (GATA-3) immunostain (×20): diffuse nuclear positivity. (**f**) Growth hormone (GH) immunostain (×20): diffuse cytoplasmic positivity. (**g**) Prolactin (PRL) immunostain (×20): patchy cytoplasmic positivity. (**h**) Cytokeratin 18 (CK18) immunostain (×20): perinuclear staining with scattered fibrous bodies

**Fig. 2:**
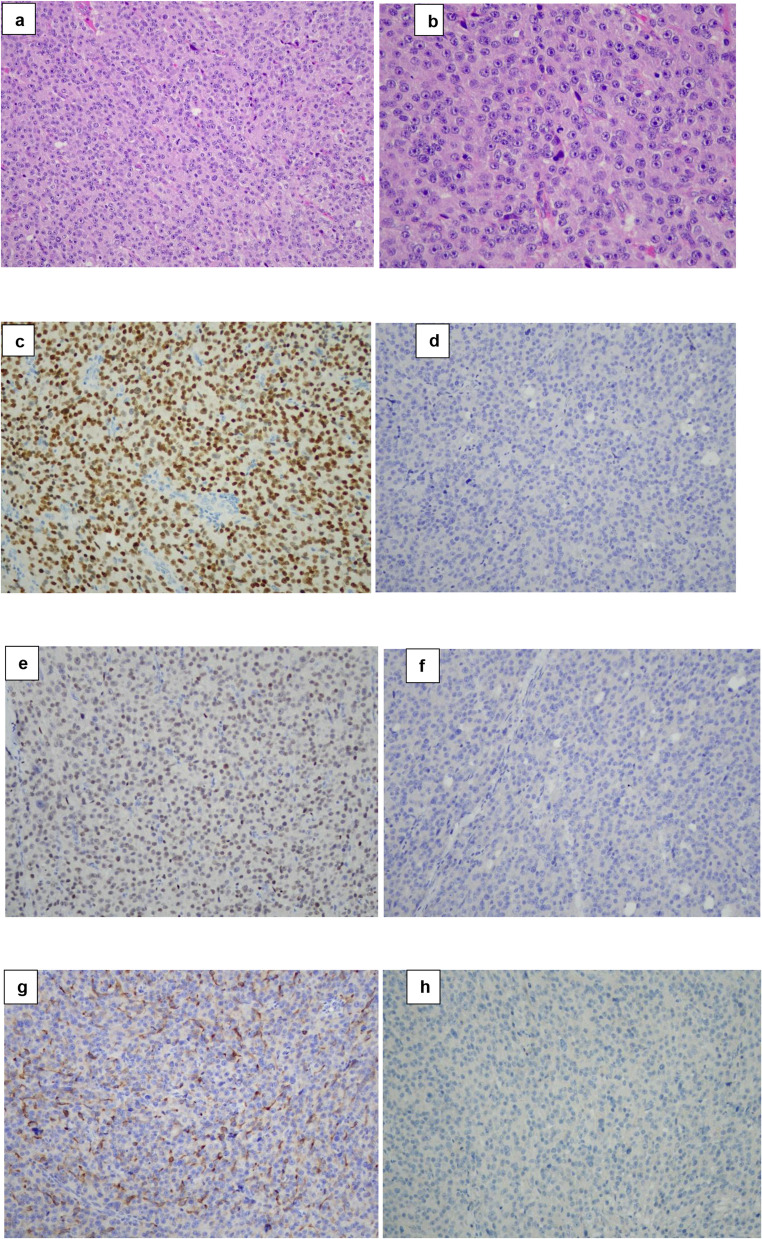
Representative histopathological and immunohistochemical features of the intracranial seeding lesion (Case 11). (**a**) Hematoxylin and eosin (H&E) (×20): solid and syncytial growth pattern composed of tumor cells with amphophilic cytoplasm. (**b**) H&E (×40): marked nuclear pleomorphism with prominent macronucleoli; a mitotic figure is identified. (**c**) Pituitary-specific positive transcription factor 1 (PIT-1) immunostain (×20): diffuse nuclear positivity. (**d**) Estrogen receptor (ER) immunostain (×20): negative. (**e**) GATA binding protein 3 (GATA-3) immunostain (×20): nuclear positivity. (**f**) Growth hormone (GH) immunostain (×20): negative. (**g**) Prolactin (PRL) immunostain (×20): positive, with predominantly cytoplasmic staining. (**h**) Thyroid-stimulating hormone (TSH) immunostain (×20): negative

Representative H&E and immunohistochemical images from Case 3, illustrating the characteristic morphological features of immature PIT-1 lineage pituitary adenomas, are presented in Fig. 1. Figure 2 presents images from the resected frontal lobe seeding lesion of Case 11, demonstrating the histomorphological and immunohistochemical features of advanced biological escalation.

The distinction from mature plurihormonal PIT-1 lineage pituitary adenomas relies on the combination of morphological immaturity, immunohistochemical discordance, and cytologic atypia, rather than hormone expression pattern alone. Although diffuse GH and/or PRL positivity was observed in Cases 3, 5, and 12, these cases were classified as immature PIT-1 lineage pituitary adenomas based on the integration of morphological and immunohistochemical features. In Case 3, despite diffuse GH and PRL positivity, ER-alpha and GATA-3 were negative, fibrous bodies were present in only 30% of cells, and prominent macronucleoli with moderate cytologic atypia were identified, a profile inconsistent with mature plurihormonal pituitary adenomas. In Case 5, GH positivity was patchy and PRL positivity, though more diffuse, was not matched by ER-alpha expression in terms of extent or intensity. Although fibrous body content was high (80%), the combination of patchy rather than diffuse GH expression, absent ER-alpha, and the presence of cytologic atypia with prominent nucleoli is inconsistent with a sparsely granulated somatotroph phenotype, collectively arguing against a mature PIT-1 lineage classification. In Case 12, GH positivity was diffuse but PRL expression was focal, ER-alpha was absent, keratin was largely negative, and fibrous bodies accounted for only 10% of cells. The morphology comprised pale eosinophilic to amphophilic cytoplasm, prominent nucleoli, sparse intranuclear inclusions, and focally spindled cells arranged in solid sheets, consistent with immature PIT-1 lineage classification

### Postoperative follow-up

Patients’ clinical characteristics, their postoperative treatment responses, and their follow-up outcomes are detailed in Table 4. For the entire cohort, the median follow-up period after surgery was 16 months (IQR: 12.5–27.5 months; overall range: 4–51 months).

**Table 4:** Clinical characteristics, treatment responses, and follow-up outcomes of the patients

Patient No	Age, Sex	Biochemical Evidence	Post-op MRI Residual Tm	Post-op Hormonal Residual	Recurrence	Current Residual Disease	Re-operation	Medical Treatment	Radiotherapy	Follow-up (months)
**1**	34, F	NF	no	no	no	no	no	no	no	25
**2**	68, F	NF	no	no	no	no	no	no	no	13
**3**	52, M	GH	no	residual	no	yes	no	Cabergoline	no	26
**4**	38, F	GH - PRL	no	no	no	no	no	no	no	37
**5**	28, F	GH	no	no	no	no	no	no	no	8
**6**	40, F	GH	no	no	no	no	no	no	no	15
**7**	39, M	NF	no	no	no	no	no	no	no	4
**8**	39, M	NF	yes (25 mm)	no	no	yes	yes	no	yes	29
**9**	48, F	GH	no	no	yes	no	yes	no	no	12
**10**	46, M	NF	yes (42 mm)	no	no	yes	yes	no	yes	18
**11**	39, F	TSH	yes (24 mm)	no	no	yes	yes	Cabergoline	yes	51
**12**	73, F	GH	yes (24 mm)	no ^a^	no	yes	no	no	no	15
**13**	57, M	TSH	no	residual	no	yes	no	Octreotide LAR	no	16

**Abbreviations: F**, female; **M**, male; **GH**, growth hormone; **TSH**, thyroid-stimulating hormone; **PRL**, prolactin; **NF**, non-functioning; **Post-op**, postoperative; **tm**, tumor; **MRI**, magnetic resonance imaging; **LAR**, long-acting release

ᵃCase 12: Early postoperative IGF-1 was mildly elevated (1.1× upper limit of normal) without clinical acromegaly; planned cabergoline was not initiated. Spontaneous IGF-1 normalization and radiological regression observed at follow-up

The initial post-surgical assessment of remission, utilizing hormonal and radiological evaluations, revealed that 46.2% (6 of 13) of patients exhibited residual disease. Of these, 4 patients had radiological residual disease and 2 patients had isolated hormonal residual disease. Adjuvant radiotherapy was administered to 3 of 13 patients, all of whom had radiological residual disease (Cases 8, 10, and 11). The two patients (Cases 3 and 13) with isolated hormonal residual disease are currently receiving medical therapy. One patient (Case 12) presented with mildly elevated IGF-1 (1.1×ULN) without overt clinical features of acromegaly in the early postoperative period; planned medical therapy was not initiated prior to follow-up. At subsequent evaluation, hormonal remission was achieved with spontaneous tumor regression from 24 mm to 17 mm (29.2% reduction), and the patient is currently under active surveillance.

Among the residual disease cases, 4 (66.7%) featured giant tumor characteristics, specifically a maximum diameter greater than 4 cm. Cavernous sinus invasion showed 3 patients with Knosp grade 4 and 3 patients with Knosp grade 2. Five patients demonstrated suprasellar extension, while infrasellar extension into the sphenoid sinus was noted in 4 patients. On T2-weighted MRI, a hyperintense signal was observed in 4 patients.

Postoperatively, a complete remission was noted in 7 patients (53.8%), all of whom exhibited normal biochemical values and no signs of tumors on imaging. Among the patients who achieved remission, one (Case 9) developed recurrence 3 years after the initial surgery; the clinical details of this case are described in the representative cases section.

Throughout the follow-up, reoperation was required in 4 patients (30.8%) due to tumor recurrence or residual disease. One acromegaly patient (case 9) developed medical treatment resistance to lanreotide and required secondary surgery. Case 11 underwent four surgical procedures in total; after the third operation, octreotide LAR was administered but failed to control the disease due to medical treatment resistance, thereby necessitating a fourth surgery. Radiotherapy (RT) was given to 3 patients (23.1%), including stereotactic radiosurgery for 2 patients (15.4%) and conventional RT for 1 patient (7.7%). Case 11 was given conventional radiotherapy for the pituitary fossa and subsequently received fractionated RT to the frontal lobe region after the seeding lesion in that area had been resected.

### Representative cases

The following three cases illustrate key clinical challenges associated with immature PIT-1 lineage tumors: delayed diagnosis (Case 10), medical treatment resistance (Case 9), and atypically aggressive behavior including intracranial seeding (Case 11).

Case 9: A patient with acromegaly, initially managed at an outside institution, experienced biochemical recurrence three years after an initially successful transsphenoidal surgery. Initial histopathological examination of the primary tumor had revealed a PIT-1 positive adenoma with focal expression of GH and PRL. The proliferation markers were low, with a Ki-67 index of 2%, a mitotic count of 1 per 2 mm², and negative pan-cytokeratin staining. Despite five years of subsequent treatment with lanreotide (90 mg every 28 days), no biochemical response was achieved. Consequently, the patient was referred to our center and underwent repeat surgical intervention. Histopathological and immunohistochemical evaluation of the second surgical specimen resulted in a diagnosis of immature PIT-1 lineage pituitary adenoma.

Case 10: The tenth patient initially underwent pituitary surgery at an external center in 2019, where the histopathological diagnosis was reported as a null cell adenoma. However, when the patient underwent repeat transsphenoidal surgery for a residual tumor three years later at our institution, subsequent immunohistochemical analysis revealed an immature PIT-1 lineage pituitary adenoma. Postoperatively, adjuvant radiotherapy was administered for the giant residual tumor.

Case 11: The patient had a complex clinical course, undergoing four pituitary-directed surgeries via transsphenoidal and transcranial approaches due to persistent disease and resistance to somatostatin analogs (octreotide LAR). Biochemically, central hyperthyroidism was present prior to the first and fourth surgeries, whereas the patient was euthyroid before the second, third, and fifth procedures. The tumor demonstrated a dynamic immunohistochemical profile across sequential surgeries. The initial pathology revealed a PIT-1(+) lineage with 70% PRL positivity and focal TSH expression. By the second and third procedures, TSH expression was lost, while PRL positivity remained dominant. Notably, the Ki-67 proliferation index increased progressively, reaching 5% by the third surgery.

The patient was referred to our center prior to the fourth surgery. The fourth surgical specimen showed a Ki-67 index of 15% and a mitotic count of 8 per 2 mm², leading to the definitive diagnosis of immature PIT-1 lineage pituitary adenoma. Following the fourth procedure, conventional radiotherapy was administered to the sellar region. After a three-year period, the patient developed a frontal lobe mass. Histopathological examination of the resected lesion confirmed intracranial seeding, with a further increase in proliferative activity: Ki-67 index reached 18% and the mitotic count rose to 15 per 2 mm². Subsequently, the patient received fractionated radiotherapy targeting the frontal lobe region.

## Discussion

This study constitutes one of the comprehensive single-center series of immature PIT-1 lineage tumors reported to date. This subtype accounted for 4% of all surgically treated pituitary adenomas at our institution over a three-year period. Our findings confirm the aggressive clinical behavior and substantial therapeutic challenges associated with this entity. During a median follow-up of 16 months, we observed a 46.2% residual disease rate and a 30.8% re-operation rate, with three cases demonstrating distinct clinical challenges as described in the representative cases section.

The 4% incidence rate in our surgical cohort is consistent with the rarity of immature PIT-1 lineage tumors, though slightly higher than previously reported frequencies ranging from 0.9% to 3.2% [4, 11, 12]. This variability likely reflects differences in referral patterns to tertiary centers, variations in immunohistochemical screening protocols, and increased recognition following the formal inclusion of this entity in the WHO 2022 classification. Our relatively higher rate likely reflects both the tertiary nature of our center and routine application of comprehensive TF immunoprofiling.

The median age at diagnosis of 37 years (range: 25–73) aligns closely with previous reports [4, 11, 13], confirming that immature PIT-1 lineage tumors can present across a broad age spectrum despite a tendency toward younger ages. We observed a notable female predominance with a female-to-male ratio of 1.6:1 (8 females, 5 males), consistent with the majority of previous series [4, 13].

One of the most striking findings of our study is the high prevalence of hormone hypersecretion, observed in 61.5% (8/13) of patients. 5 with acromegaly (38.5%), 2 with TSH-secreting tumors (15.4%), and 1 with GH-PRL co-secretion (7.7%). Only 38.5% (5/13) were clinically non-functioning. This distribution contrasts markedly with Lang et al., who reported the majority of cases as non-functioning tumors [4], and Erickson et al., who similarly found that most tumors were non-functioning, with acromegaly observed in only 19% of cases [11], and Mete et al., who documented hormone hypersecretion in only 29% of cases [12]. These findings suggest that hormonally active tumors within the immature PIT1-lineage group may be more prevalent than previously recognized, and each additional case series will contribute to expanding our understanding of the true clinical spectrum of this rare entity.

Notably, we identified no tumors with isolated lactotroph differentiation. Regarding prolactin hypersecretion specifically, although PRL immunoreactivity is a common finding across all published series, clinically significant prolactinoma-like behavior has been rare and inconsistent. In the series by Erickson et al., 17 of 27 tumors (63%) demonstrated PRL immunoreactivity, and only 3 patients (11%) had PRL levels exceeding 150 ng/mL; of these, only two cases were described as clinically resembling prolactinomas [11]. In the series by Mete et al., hyperprolactinemia above 150 µg/L was documented in only one patient (4%), who was found to harbor a MEN-1 mutation [12]. In the series by Lang et al., while increased serum PRL levels were reported in 53.33% of patients, individual values and the proportion exceeding the 150 ng/mL threshold were not reported, precluding direct comparison [4].

This observation may reflect the ontogenetic hierarchy of PIT1-lineage cytodifferentiation. In the developing anterior pituitary, GH synthesis precedes PRL synthesis, a developmental sequence established in rodent models and considered consistent with human pituitary organogenesis [14, 15]. Furthermore, while PIT-1 alone can initiate basal transcription of both GH and PRL genes, somatotroph terminal differentiation is driven by PIT-1 alone, whereas full lactotroph maturation with high-level autonomous PRL secretion additionally requires synergistic co-expression of estrogen receptor-alpha with PIT-1, representing a further transcriptional commitment step [15, 16]. Tumors arrested at an immature stage of this developmental pathway, as defined by the 2022 WHO classification [2], may therefore express PRL at a limited level but lack the full transcriptional apparatus required for autonomous prolactin hypersecretion. This interpretation remains speculative, however, and requires validation through dedicated translational studies.

All patients in our cohort presented with macroadenomas, a finding uniformly observed across all published series [4, 12]. Our median tumor diameter of 28 mm (range: 15–59 mm) is consistent with previous reports. While 4 patients (30.8%) presented with giant tumors (> 4 cm), a fifth patient had a borderline maximum diameter of 39 mm; together, these 5 patients (38.5%) presented with substantially large tumors, further distinguishing our series from the rates reported by Mete et al. (9%) and Horvath et al. (27.6%) [12, 13].

Tumor extension patterns demonstrated the aggressive multicompartmental growth characteristic of this entity. Suprasellar growth was observed in 12 patients (92.3%), of whom 5 (38.5%) had optic chiasm compression with documented visual field defects, while infrasellar extension was present in 10 patients (76.9%). These findings are comparable to Lang et al., who reported suprasellar and infrasellar growth in 100% and 93.3% of cases, respectively [4]. Notably, no tumor in our series remained confined to the sella; all cases exhibited extrasellar extension, either suprasellar or infrasellar, and none were classified as Knosp grade 0. This universal pattern of extrasellar growth may be attributable to delayed clinical presentation or the inherently rapid growth kinetics of this tumor subtype. According to the Knosp classification, 5 patients (38.5%) demonstrated definitive cavernous sinus invasion (grades 3–4), consistent with Lang et al. (40%) and Erickson et al. (37%) [4, 11]. This invasion rate aligns with the 40–50% range reported in the literature for immature PIT-1 lineage tumors, consistent with the reported for invasive growth [5].

On T2-weighted MRI, the majority of tumors exhibited hyperintense signal (62%), though variable signal characteristics were observed, including isointense (23%), hypointense (8%), and mixed signal intensity (8%), reflecting heterogeneous tumor composition. While T2 hyperintensity may aid in differential diagnosis, the lack of a uniform signal pattern underscores the need for histopathological confirmation. The radiological findings in our cohort, including large tumor size, multicompartmental extension, and cavernous sinus invasion, are consistent with the aggressive biological behavior described for this entity in the WHO 2022 classification.

On histopathological examination, all tumors demonstrated universal PIT-1 nuclear positivity, confirming pure PIT-1 lineage differentiation. Notably, three patients were diagnosed with immature PIT-1 lineage tumors only after repeat surgery for recurrent or residual disease. On immunohistochemical analysis, the majority of tumors (77%) exhibited a plurihormonal profile with GH predominance. These findings are largely consistent with previous reports, including Lang et al. (GH 80%, PRL 93%) and Erickson et al. (74% plurihormonal) [4, 11], supporting that plurihormonality is a hallmark feature reflecting the origin from incompletely differentiated progenitor cells capable of multilineage hormone production. Our lower TSH immunostaining rate (30.8%) compared to Lang et al. (73%) [4] may reflect variability in immunohistochemical techniques, antibody sensitivity, or true biological heterogeneity.

GATA-3 positivity was observed in 6 cases (46.2%), while ER showed variable positivity in 5 cases (38.5%). Notably, both TSH-secreting tumors in our series (Cases 11 and 13) demonstrated GATA-3 expression, consistent with its characteristic association with thyrotroph differentiation [2, 17]. The variable GATA-3 positivity observed likely reflects differing degrees of partial thyrotroph commitment within this heterogeneous tumor category.

Proliferative markers demonstrated significant heterogeneity. The median Ki-67 index was 4% (IQR: 2–10%; range: 1–35%), with 53.8% (7/13) of cases demonstrating Ki-67 ≥ 3%, a threshold associated with increased proliferative activity. Notably, 23.1% (3/13) of cases exhibited Ki-67 ≥ 10%, meeting the criteria for “highly proliferative” tumors (grade 2b*) according to the five-tiered clinicopathological classification [18]. These findings are consistent with Mete et al. (mean 4%) and Erickson et al. (up to 9%) [11, 12], though the wide range indicates significant biological heterogeneity. The median mitotic count was 4 per 2 mm² (IQR: 1–8; range: 1–20), notably higher than Lang et al.‘s median of < 1 per 10 HPF [4]. When considering the combination of universal extrasellar extension and elevated proliferative markers in our cohort, the majority of cases would qualify as grade 2b tumors, defined by the co-occurrence of radiological invasiveness and proliferative activity, which have been proposed to represent ‘tumors with malignant potential [18]. The concordance between moderate Ki-67 indices and correspondingly elevated mitotic activity suggests may suggest a higher proliferative potential in immature PIT-1 lineage tumors compared to other pituitary adenoma subtypes. These features, combined with universal cytologic atypia, support the WHO 2022 classification’s emphasis on histological subtype classification for prognostic assessment rather than relying solely on proliferation markers [2].

Cytokeratin-18 expression was present in 76.9% of cases, predominantly with focal staining patterns, while fibrous bodies were identified in 46.2% of cases, occupying a median of 20% of tumor volume (range: 10–80%), notably higher than the 16% reported by Mete et al. [12]. Assessment of fibrous bodies is particularly relevant in the differential diagnosis with sparsely granulated somatotroph pituitary adenomas, in which fibrous bodies are characteristically diffuse and present in more than 70% of tumor cells per WHO 2022 criteria. In our cohort, even in cases with acromegaly and diffuse GH positivity, fibrous body content remained well below this threshold, supporting the immature PIT-1 lineage classification over a sparsely granulated somatotroph phenotype. Notably, one case (Case 5) demonstrated 80% fibrous body content; however, GH positivity was patchy rather than diffuse, PRL positivity was present, ER-alpha was absent, and cytologic atypia with prominent nucleoli was identified, collectively arguing against a sparsely granulated somatotroph designation.

One patient (14.3%, 1/7) subsequently developed recurrence during follow-up. This was a female patient with acromegaly who initially achieved biochemical and radiological remission after 3 years but later developed tumor regrowth requiring repeat intervention. Erickson et al. similarly reported a 15% recurrence rate in their series [11]. While our observed recurrence rate of 14.3% with limited follow-up (median 16 months) appears modest, previous studies with longer follow-up demonstrate substantially higher recurrence rates for immature PIT-1 lineage tumors, with up to 50% recurrence risk at 10 years [2, 4], in stark contrast to mature PIT-1 lineage tumors which demonstrate near-zero recurrence post-remission [5, 19]. Our relatively short follow-up duration precludes definitive conclusions regarding long-term recurrence risk, and extended surveillance will be essential to determine the true recurrence burden in this cohort.

The re-operation rate of 30.8% exceeds the typical 10–15% reported for unselected pituitary adenoma cohorts [20, 21]. Case 9 underwent reoperation following tumor recurrence and resistance to lanreotide therapy, while the remaining three patients required repeat surgery due to significant residual tumor burden with maximum diameters reaching up to 4 cm. This elevated re-operation rate most likely reflects the inherently invasive growth pattern of these tumors and the high prevalence of giant adenomas in our series; although surgical technique and experience may also influence resection outcomes, all procedures were performed by dedicated pituitary neurosurgeons at a tertiary referral center.

Medical treatment resistance was observed in two patients with GH-secreting tumors in our cohort. Case 9 demonstrated complete resistance to lanreotide despite five years of continuous treatment, ultimately requiring reoperation, and Case 11 failed to respond to octreotide LAR following the third surgical procedure, necessitating a fourth surgery. These observations are consistent with the expected weak, heterogeneous, or absent somatostatin receptor (SSTR) expression, particularly SSTR2 and SSTR5, reported in immature PIT-1 lineage tumors [22, 23]. Nevertheless, two patients with residual disease (Cases 3 and 13) are currently maintained on medical therapy with close surveillance. These findings suggest that while medical management may have a role in selected cases, early consideration of radiotherapy or alternative therapies may be prioritized over prolonged ineffective somatostatin analogue trials.

Adjuvant radiotherapy was administered to 3 (50%) of the 6 patients with residual disease, representing 23.1% of our entire cohort. Among these, two patients received stereotactic radiosurgery, while one received conventional radiotherapy. This rate is considerably lower than the 54% reported by Erickson et al. [11], where the majority of patients required external beam irradiation due to tumor persistence. The relatively lower rate of adjuvant radiotherapy in our series compared to previously published cohorts may reflect differences in treatment approach, though the small sample size precludes definitive conclusions.

In the two patients who underwent radiotherapy for residual disease, no increase in tumor size was observed during the post-radiotherapy follow-up period. However, the efficacy of radiotherapy in preventing aggressive disease progression remains uncertain, as evidenced by Case 11, who developed intracranial seeding three years after conventional radiotherapy despite initial radiological stability. Given the high rates of residual disease (46.2%), potential long-term recurrence risk (up to 50% at 10 years), and medical treatment resistance characteristic of immature PIT-1 lineage tumors, the optimal timing and indications for adjuvant radiotherapy warrant further investigation. Potential high-risk features that may justify earlier consideration of adjuvant radiotherapy include incomplete resection with significant residual tumor, advanced cavernous sinus invasion (Knosp grade 3–4), elevated Ki-67 proliferation index (≥ 3–5%), or progressive disease despite medical therapy. Prospective studies with extended follow-up are needed to determine whether early adjuvant radiotherapy can reduce long-term recurrence rates and minimize the need for multiple surgical interventions that may increase the risk of iatrogenic complications.

Case 11 exemplifies iatrogenic tumor seeding, a rare but well-documented complication in aggressive pituitary adenomas. A systematic review by Azizkhanian et al. identified 136 cases of distant pituitary adenoma spread, with 98.5% occurring after surgical resection and a median latency of 96 months, with craniotomy representing a prominent risk factor compared to transsphenoidal approaches [6, 24, 25]. Our patient underwent four surgical interventions via repeated transcranial approaches, and three years after the fourth surgery and adjuvant radiotherapy, a frontal lobe mass was confirmed histopathologically as tumor seeding. Serial resections demonstrated progressive biological escalation, with Ki-67 indices rising from 5% to 18%, immunohistochemical dedifferentiation, and a fluctuating hormonal course reflecting retained capacity for autonomous TSH secretion. This fluctuating secretory phenotype across successive recurrences, together with rising proliferative indices and intracranial dissemination, likely reflects the clonal heterogeneity and biological instability inherent to immature PIT1-lineage tumors, rather than the more predictable behavior of classical thyrotroph adenomas [25]. This case underscores the necessity for prolonged surveillance extending well beyond conventional intervals, even in patients demonstrating apparent radiological stability. The three-year latency period and progressive dedifferentiation observed across serial resections support strategies to minimize repeat surgeries through aggressive initial resection when feasible and early consideration of adjuvant radiotherapy for incompletely resected tumors.

Case 10 highlights a critical diagnostic pitfall: the tumor was initially diagnosed as a null cell adenoma at an external center, and the correct diagnosis of immature PIT-1 lineage pituitary adenoma was only established three years later at our institution through comprehensive TF immunohistochemistry. This case underscores the importance of systematic TF immunostaining in all pituitary adenomas, particularly those lacking obvious hormone expression.

Overall, the TF-based classification system must be interpreted in conjunction with morphological features (cytologic atypia, proliferative markers) and clinical behavior to guide appropriate management. We recommend centralized pathology review for all suspected cases of aggressive pituitary tumors to ensure accurate diagnosis and consistent application of WHO 2022 criteria. The variability in clinical presentation, ranging from apparently silent tumors to those with overt hormone hypersecretion, further underscores the critical importance of histopathological diagnosis rather than relying solely on clinical phenotype.

Treatment strategy should prioritize maximal safe surgical resection as the primary therapeutic modality, given the frequent resistance to medical therapy observed in this entity. For patients requiring multiple surgeries, careful consideration should be given to surgical approach selection. Endoscopic transsphenoidal techniques should be preferred when anatomically feasible to minimize the risk of tumor cell dissemination, as demonstrated in Case 11 following repeated transcranial approaches. In cases with advanced cavernous sinus invasion (Knosp grade 3–4), giant tumors (> 4 cm), or features suggesting incomplete resectability, early consideration of adjuvant radiotherapy may be warranted rather than pursuing multiple surgical procedures with cumulative risks. Medical therapy with somatostatin analogues should not be relied upon as primary treatment given the expected low SSTR2/SSTR5 expression in these tumors; however, a trial may be considered in select cases with careful monitoring for treatment response.

Patients with immature PIT-1 lineage tumors require intensive long-term surveillance with regular biochemical and radiological assessments. Given the high residual disease rate and recurrence potential, close follow-up with pituitary MRI and hormonal evaluation is essential, with additional whole-brain imaging in patients who have undergone craniotomy. A multidisciplinary team approach involving endocrinologists, neurosurgeons, radiation oncologists, and specialized pituitary pathologists is essential for optimal management.

Several limitations should be acknowledged. The retrospective single-center design limits causal inference, and the cohort of 13 patients, though comparable to other published series (Horvath et al.: 29 patients; Erickson et al.: 27 patients; Mete et al.: 25 patients; Lang et al.: 15 patients), constrains statistical power. Eight patients (38%) were excluded due to loss to follow-up; however, baseline characteristics did not differ significantly from the analyzed cohort, although the small group sizes limit the statistical power of this comparison and selection bias cannot be definitively excluded. Multivariable survival analysis was not feasible given the small sample size and limited recurrence events. The median follow-up of 16 months, with a minimum of 4 months in a subset of patients, is insufficient to fully characterize long-term recurrence patterns, and conclusions should be considered preliminary given that recurrence rates of up to 50% at 10 years have been reported. Molecular characterization including SSTR2/SSTR5 expression analysis was not systematically performed. Formal grading of cytologic atypia and systematic assessment of stromal fibrosis were not feasible within the retrospective design, as both would require a dedicated histopathological slide-review study beyond the scope of this clinical outcome analysis; these represent important parameters for future prospective investigations with centralized pathology review. Given these constraints, our findings should be considered exploratory and require validation in larger, multicenter cohorts with extended follow-up, which will also be essential for accumulating sufficient case numbers to refine prognostic criteria and optimize outcomes for this rare entity.

In conclusion, immature PIT-1 lineage pituitary adenomas represent a distinct and clinically aggressive subset of pituitary adenomas that demand heightened awareness and specialized management. Our findings are consistent with the WHO 2022 classification of these tumors as high-risk neoplasms, characterized by large size, high residual disease rates, and potential for rare complications including intracranial seeding with medical treatment resistance observed in a subset of patients. Accurate diagnosis through routine comprehensive TF immunohistochemistry is paramount for proper risk stratification and treatment planning. Management should prioritize maximal safe surgical resection, with early consideration of adjuvant radiotherapy for high-risk features, and intensive long-term surveillance extending beyond 10 years given the potential for late recurrence. Multicenter collaboration will be essential for refining prognostic criteria, identifying molecular predictors, and optimizing outcomes for patients with these challenging neoplasms.

## Supplementary Information

Below is the link to the electronic supplementary material.

Supplementary File 1 (DOCX 16.8 KB)

## Data Availability

No datasets were generated or analysed during the current study.
