# Supplementary material for: Clinicopathological features and outcomes of immature PIT-1 lineage tumors: A high-risk pituitary neuroendocrine tumor/pituitary adenoma subtype per WHO 2022
Source: Pituitary. 2026 May 13;29(3):84. doi: 10.1007/s11102-026-01691-9 (PMC13171669; doi:10.1007/s11102-026-01691-9)
Supplement: Supplementary file 1 — (DOCX 16.8 KB) [file 11102_2026_1691_MOESM1_ESM.docx]

**Supplementary Table 1.** Baseline Clinicopathological Characteristics of Analyzed Cohort (n = 13) Versus Excluded Patients (n = 8)

| **Characteristic** | **Analyzed Cohort (n = 13)** | **Excluded Patients (n = 8)** | **p-value** |
| --- | --- | --- | --- |
| **Demographics** | | | |
| Age (years) |  |  |  |
| Median (IQR) | 37 (28.5–54.5) | 52 (27–58) | > 0.05 |
| Range | 25–73 | 25–63 |  |
| Sex, n (%) |  |  | > 0.05 |
| Female | 8 (61.5) | 6 (75.0) |  |
| Male | 5 (38.5) | 2 (25.0) |  |
| **Tumor Characteristics** | | | |
| Tumor size (mm) |  |  |  |
| Median (IQR) | 28 (16–41) | 11.5 (9.2–12.0) | > 0.05 |
| Range | 15–59 | 6–30 |  |
| Knosp grade, n (%) |  |  | > 0.05 |
| Grade 1–2 | 8 (61.5) | 3 (75.0)ᵃ |  |
| Grade 3–4 | 5 (38.5) | 1 (25.0)ᵃ |  |
| Ki-67 index (%) |  |  |  |
| Median (IQR) | 4 (2–10) | 2.0 (1.8–6.2) | > 0.05 |
| Range | 1–35 | 1–9 |  |
| Mitotic count (/10 HPF) |  |  |  |
| Median (IQR) | 4 (1–8) | 2.0 (1.0–3.5)ᵇ | > 0.05 |
| Range | 1–20 | 0–7ᵇ |  |
| **Hormonal Phenotype** | | | |
| Acromegaly, n (%) | 5 (38.5) | 5 (62.5) | > 0.05 |
| GH + PRL co-secretion, n (%) | 1 (7.7) | 1 (12.5) |  |
| TSH-secreting (TSHoma), n (%) | 2 (15.4) | 1 (12.5) |  |
| Non-functioning, n (%) | 5 (38.5) | 1 (12.5) |  |

Abbreviations: GH, growth hormone; PRL, prolactin; TSH, thyroid-stimulating hormone; IQR, interquartile range; HPF, high-power field.

ᵃ Knosp grading data available for 4 of 8 excluded patients (50%). ᵇ Mitotic count data available for 7 of 8 excluded patients (87.5%).

p-values calculated using Mann–Whitney U test for continuous variables and chi-square or Fisher's exact test for categorical variables. No statistically significant differences were observed between groups (all p > 0.05).
